# LONELINESS AND MEMORY LAPSES AMONG OLDER ADULTS IN DAILY LIFE

**DOI:** 10.1093/geroni/igae098.2215

**Published:** 2024-12-31

**Authors:** Karina Van Bogart, Erin Harrington, Jacqueline Mogle, Jee eun Kang, Martin Sliwinski, Jennifer Graham-Engeland

**Affiliations:** Northwestern University, Chicago, Illinois, United States; University of Wyoming, Laramie, Wyoming, United States; Clemson University, Clemson, South Carolina, United States; The Pennsylvania State University, University Park, Pennsylvania, United States; Pennsylvania State University, University Park, Pennsylvania, United States; Pennsylvania State University, University Park, Pennsylvania, United States

## Abstract

Loneliness has been linked to objective cognitive decline and dementia risk; however, less is known about how loneliness relates to subjective cognition. Assessing daily subjective cognition related to prospective memory (i.e., for future events; PM) and retrospective memory (i.e., for past information; RM) may be particularly useful in identifying associations with daily emotional and cognitive health. The current research examined associations between loneliness and self-reported PM and RM lapses among 311 older adults (mean age=77.45 years, 40% Black, 13% Hispanic, 67% women) who completed 14 consecutive days of ecological momentary assessment that included self-reports of momentary loneliness (5x/day) as well as evening self-reports of memory lapses that occurred that day. Separate multilevel models were estimated to assess daily associations between loneliness and number of PM or RM lapses, and the amount of disruption and irritation associated with these lapses. All models controlled for age, gender, years of education, MCI status, and depressive symptoms. Days when loneliness was higher than typical were significantly associated with a higher number of PM lapses (b=0.011, p<.001), and more disruption to daily activities (b=0.113, p <.001) and irritation (b=0.036, p=.002) associated with experiencing PM lapses. Daily loneliness was not associated with the number of RM lapses, but was significantly linked with more disruption to daily activities (b=0.131, p<.001) and irritation associated with RM lapses (b=0.103, p<.001). Findings suggest that loneliness may uniquely impact daily PM and perceived disruption from RM issues, which has important implications for cognitive health and maintaining independence in late life.

